# T-BAS Version 2.1: Tree-Based Alignment Selector Toolkit for Evolutionary Placement of DNA Sequences and Viewing Alignments and Specimen Metadata on Curated and Custom Trees

**DOI:** 10.1128/MRA.00328-19

**Published:** 2019-07-18

**Authors:** Ignazio Carbone, James B. White, Jolanta Miadlikowska, A. Elizabeth Arnold, Mark A. Miller, Nicolas Magain, Jana M. U'Ren, François Lutzoni

**Affiliations:** a Center for Integrated Fungal Research, Department of Entomology and Plant Pathology, North Carolina State University, Raleigh, North Carolina, USA; b Department of Biology, Duke University, Durham, North Carolina, USA; c School of Plant Sciences, The University of Arizona, Tucson, Arizona, USA; d Department of Ecology and Evolutionary Biology, The University of Arizona, Tucson, Arizona, USA; e San Diego Supercomputer Center, University of California, San Diego, La Jolla, California, USA; f Institut de Botanique B22, Biologie de l’Évolution et de la Conservation, UR InBios, Université de Liège, Liège, Belgium; g Department of Biosystems Engineering, The University of Arizona, Tucson, Arizona, USA; Broad Institute

## Abstract

The Tree-Based Alignment Selector (T-BAS) toolkit combines phylogenetic-based placement of DNA sequences with alignment and specimen metadata visualization tools in an integrative pipeline for analyzing microbial biodiversity. The release of T-BAS version 2.1 makes available reference phylogenies, supports multilocus sequence placements and permits uploading and downloading trees, alignments, and specimen metadata.

## ANNOUNCEMENT

**FIG 1:**
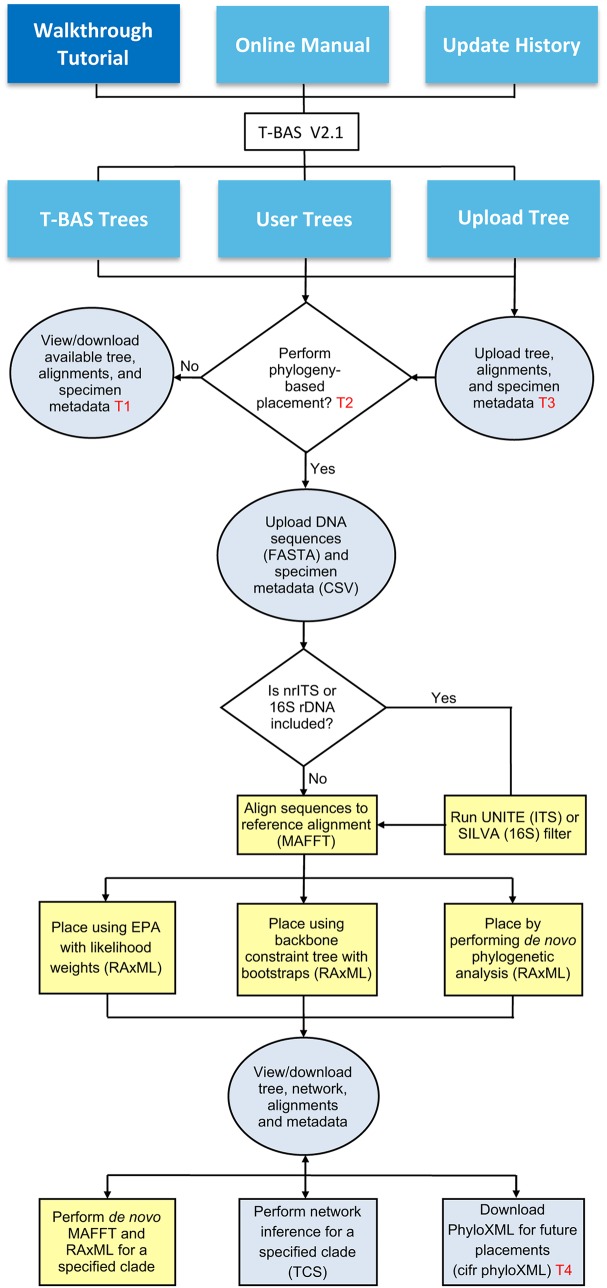
A schematic flowchart showing the three major features in T-BAS version 2.1 and their partitioning between servers at North Carolina (NC) State University (blue) and CIPRES (yellow). Reference trees included in this release are fungi (22), bacteria (23), Pezizomycotina version 1 (1) and extended version 2, Lecanoromycetes (24), Laboulbeniomycetes (25), Sebacinales (26), Xylariaceae (27), *Peltigera* (28), *Ramularia eucalypti* species complex (29), *Aspergillus* section *Flavi* (7), and the *Fusarium* species complex (30). The three primary user actions (gray) are (i) select a T-BAS reference tree, which includes DNA sequence alignments and specimen metadata information for viewing and downloading; (ii) perform phylogeny-based placement of DNA sequences for up to seven loci on a T-BAS tree, directly or after sequence data are compiled into operational taxonomic units (OTUs); or (iii) upload a new tree with alignments and metadata and place specimen sequences, with the option of submitting it for inclusion in the T-BAS framework. Documentation includes tutorials, a user manual, and an update history which lists the new features in T-BAS version 2.1 compared with those in version 1.0. Abbreviations in the flowchart are as follows: T1, tutorial 1; T2, tutorial 2; T3, tutorial 3; T4, tutorial 4; EPA, Evolutionary Placement Algorithm (17); UNITE, unified system for the DNA-based fungal species linked to the classification (19); SILVA, database for rRNA gene sequences (16S rDNA) (20); BLAST, Basic Local Alignment Search Tool (31); RAxML, Randomized Axelerated Maximum Likelihood (13); MAFFT, Multiple Alignment using Fast Fourier Transform (32); PhyloXML, standard using extensible markup language (8) to encode phylogenetic tree, sequence alignments, and specimen metadata information.

The Tree-Based Alignment Selector (T-BAS) toolkit offers an integrated phylogenetic analysis and visualization framework that combines evolutionary placement of DNA sequences with multilocus sequence alignments and metadata (e.g., countries, states, sites, hosts, sequence data, taxonomic classifications, and phenotypic traits), enabling biodiversity discovery and facilitating rapid species description (1–3). Here, we announce the public release of T-BAS version 2.1, which builds on the functionality of T-BAS version 1.0 and allows users to upload any reference tree, multilocus DNA sequence alignments, and specimen metadata and to perform multilocus placements of DNA sequences with user-customizable metadata (Fig. 1). Phylogeny-based placement of DNA sequences can be performed on trees and multilocus alignments (e.g., those available in TreeBase [www.treebase.org] [4, 5] or custom trees uploaded by users), greatly expanding the utility of T-BAS across biological disciplines. Trees, alignments, and specimen metadata also can be uploaded and viewed without performing placements (6, 7), making it a versatile phylogenetic tool across diverse taxonomic groups.

In T-BAS version 2.1, phylogenetic placement and specimen metadata output are standardized via the extended PhyloXML format (8) so that placements can be readily viewed by programs such as PhyD3 (9), IcyTree (10), TreeGraph 2 (11), and Archaeopteryx.js (version 0.9928 beta-2018-07-05) (12). Metadata can be displayed as colorized outer rings in phylogenies, as network graphs with node attributes, or as pie charts via the JavaScript library D3.js (https://d3js.org/). Users can select specific clades on tree outputs from T-BAS analyses and then perform phylogenetic inference across single or multiple alignments with RAxML (13) or network inference with the genealogical method of Templeton et al. (14) implemented in TCS version 1.21 (15) and NetworkX (16). Three phylogeny-based placement options are available, including Evolutionary Placement Algorithm (EPA) (17), backbone constraint tree, and *de novo* phylogenetic reconstruction via RAxML (13) through the Representational State Transfer Application Program Interface (REST API) service at Cyberinfrastructure for Phylogenetic Research (CIPRES) (18). Synchronization of color-coded attributes across subtrees and networks is possible using a new specimen metadata color editor (see online user manual).

A new guide tree framework in T-BAS version 2.1 allows users to select the appropriate reference tree for multilocus placements of taxa via sequence data for up to seven loci. Phylogenomic trees based on hundreds of loci also can serve as reference trees for multilocus placement of DNA sequences. Prefiltering of barcode sequences is included as an option via the UNITE fungal nrITS database (version 7.2 release date, 28 June 2017) (19) and SILVA bacterial database (release version 132) (20). This is important when placing sequences from DNA metabarcoding of nrITS for organisms such as fungi to ensure that only taxon sequences within the taxonomic breadth of the reference trees are aligned.

Fine resolution of phylogenetic relationships is possible in T-BAS version 2.1 via an expanded phylogenetic framework that can accommodate more reference trees and increased taxonomic sampling. For example, the Pezizomycotina reference tree released in T-BAS version 1.0 has been extended from 979 to 1,625 taxa based on NCBI RefSeq records (21) and provides access to alignments for up to six loci (nr5.8S, nrLSU, nrSSU, mtSSU, *RPB1*, and *RPB2*). Additional reference trees, multilocus alignments, and specimen metadata are made available (Fig. 1). We anticipate the number of trees available for placement to grow as users contribute new reference trees and metadata. T-BAS version 2.1 allows the research community to build on phylogenetic resources by submitting reference trees for inclusion in the T-BAS framework and sharing these trees, alignments, and specimen metadata with other worldwide experts to enhance taxonomic discovery, description of new species, and the tracking of biodiversity for ecological studies.

### Data availability.

The T-BAS toolkit is available at https://tbas.hpc.ncsu.edu/.
